# Quantifiable Effect of Interparticle Plasmonic Coupling on Sensitivity and Tuning Range for Wavelength-Mode LSPR Fiber Sensor Fabricated by Simple Immobilization Method

**DOI:** 10.3390/s22239075

**Published:** 2022-11-23

**Authors:** Shuo Jia, Aiwen Ma, Hanpeng Dong, Shanhong Xia

**Affiliations:** 1 Center for Advanced Measurement Science, National Institute of Metrology, Beijing 100029, China; 2 State Key Laboratory of Transducer Technology, Aerospace Information Research Institute, Chinese Academy of Sciences, Beijing 100190, China; 3 School of Precision Instrument and Opto-Electronics Engineering, Tianjin University, Tianjin 300072, China; 4 Chinese Society for Measurement, Beijing 100029, China

**Keywords:** fiber sensor, gold nanospheres, localized surface plasmon resonance, interparticles plasmonic coupling effect, refractive index test

## Abstract

Herein a gold nanosphere (AuNS)-coated wavelength-mode localized surface plasmon resonance (LSPR) fiber sensor was fabricated by a simple and time-saving electrostatic self-assembly method using poly(allylamine hydrochloride). Based on the localized enhanced coupling effect between AuNSs, the LSPR spectrums of the AuNS monolayer with good dispersity and high density exhibited a favourable capability for refractive index (RI) measurement. Based on the results obtained from the optimization for AuNS distribution, sensing length, and RI range, the best RI sensitivity of the fiber modified by 100 nm AuNS reached up to about 2975 nm/RIU, with the surrounding RI range from 1.3322 to 1.3664. Using an 80 nm AuNS-modified fiber sensor, the RI sensitivity of 3953 nm/RIU was achieved, with the RI range increased from 1.3744 to 1.3911. The effect of sensing length to RI sensitivity was proven to be negligible. Furthermore, the linear relationship between the RI sensitivity and plasma resonance frequency of the bulk metal, which was dependent on the interparticle plasmon coupling effect, was quantified. Additionally, the resonance peak was tuned from 539.18 nm to 820.48 nm by different sizes of AuNSs-coated fiber sensors at a RI of 1.3322, which means the spectrum was extended from VIS to NIR. It has enormous potential in hypersensitive biochemistry detection at VIS and NIR ranges.

## 1. Introduction

Gold nanoparticles (AuNPs) have been used as excellent scaffolds for biochemical sensors for their unique optoelectronic properties [1]. AuNPs produce a significant visible absorption band when the input photon frequency matches the collective electron charge oscillations. These resonance properties, tuned by varying sizes and shapes of AuNPs, are known as the localized surface plasmon resonance (LSPR) [2]. LSPR sensors, which have the advantages of small sensing volume, temperature-insensitivity, and the flexible design of nanostructures, have been utilized for a broad variety of biological interactions, due to the sensitivity of nanostructures to the refractive index (RI) [3].

The optical fiber-based LSPR sensors were primarily classified into two types: intensity mode and wavelength mode [4]. They have the benefits of simplicity of construction, integration simplicity, and remote monitoring. The intensity mode is more often utilized for biological sensing than the wavelength mode, due to the latter’s lower RI sensitivity. Chau et al. firstly proposed absorbance-modulated fiber sensors for streptavidin detection with an LOD of 9.8 * 10^−11^ M. AuNS with 8.4 nm diameter were modified on unclad fibers by silane coupling agents [5]. Then, this group used this LSPR fiber sensor for antibody antigens [6], viruses [7], and ions [8]. Sai et al. fabricated an absorbance-mode U-type PMMA optical fiber RI sensor. A total of 37 nm AuNS was immobilized on the amine functionalized PMMA by hexamethylenediamine and had an RI sensitivity of about 5.57 AU/RIU [9]. Duan et al. raised a Ω-shaped fiber sensor with gold nanoparticles that were coated for MCF-7 cancer cells for rapid and ultra-sensitive detection [10].

However, for on-site measurements, the wavelength mode was preferable, since it was less subject to interference from the surrounding environment, such as changes in ambient lighting. The core parameter for the RI sensitivity (S_RI_) of wavelength-modulated LSPR fiber sensors has been studied adequately in last decade by optimizing different aspects, such as optical fiber structures [11,12,13], nanomaterials [14,15,16], and preparing methods [17,18]. Sun et al. investigated a variety of important parameters, including the size of the gold nanospheres (AuNSs), the pH of the AuNSs solutions, the AuNSs immobilizing time, and the temperature of the fiber substrates [19]. After that, the gold nanorods and gold nanocages were coated as the sensing elements in their further research [20,21]. Zhang et al. reported that the graphene and Ag nanoparticles modified the U-bend optical fiber by laser-induced deposition for RI sensing [22]. Peng et al. reported a AuNSs-modified fiber biosensor for human IgG detection by a poly(styrene-b-4-vinylpyridine) polyelectrolyte assembly method. The highest S_RI_ was 1079 nm/RIU, optimizing the size of the AuNSs [23]. In their further research, gold nanorods were modified by the same immobilization method, with a S_RI_ about 753 nm/RIU [24]. Furthermore, gold nanoparticle-coated fibers of layer-by-layer polyelectrolyte films had a S_RI_ of about 1312 nm/RIU, as the latest research in 2022 showed [25]. However, the imperfectly immobilized surface, such as the low density and aggregation of nanoparticles, confined the achievements of higher S_RI_.

The localized interparticle plasmonic coupling-enhanced effects were excited when two metal nanoparticles were placed in proximity. This closed even aggregated metal nanoparticles with a nanometer length scale, which was called a “hot-spot”, and it has been employed in surface-enhanced Raman scattering (SERS) biochemical detections [26]. In addition, the plasmonic coupling effect, which was tuned by changing the size-to-gap ratio between the metal nanoparticles, shifted the localized plasmonic resonance wavelength [27]. This tuning effect was interpreted by the attenuation of the repulsive forces between the metal nanoparticles, which led to a correspondingly lower resonance frequency and higher resonance wavelength [28]. It is very critical for improving the S_RI_ of wavelength-modulated LSPR sensors.

In light of the above, based on interparticle plasmon coupling effect between the AuNSs coated on unclad fiber sensors, our work aims to explore a wavelength-modulated LSPR fiber sensor by fabricating optimally designed plasmon configurations of interacting nanoparticles for RI measurement. The immobilized AuNSs on the surface of fiber observed by SEM were high density and of great dispersity, which means the generation of a resonance coupling effect. The key parameters, including the size, density, sensing length, and RI range, were optimized systematically by determining the best S_RI_. The interparticle coupling effect was proven as the key fact for S_RI_ by experimental data. Then, the figure of merit (FOM), consistency, and stability of the fiber sensor were also estimated in detail. Finally, the S_RI_ was evaluated and compared with other groups. To the best of our knowledge, this quantitative study for sensitivity enhancement is first to propose wavelength-mode LSPR fiber sensors. More importantly, the S_RI_ with a different RI range reached a higher level than all the previous research for noble nanoparticle-coated LSPR fiber sensors.

## 2. Principle and Experimental Details

### 2.1. Principle

The LSPR spectrum of isolated AuNS is dependent on the size, shape, and dielectric characteristics of the AuNS’s surroundings. However, for high density AuNSs, a coated silica surface for the coupling between AuNSs must be considered seriously because of its influence on the LSPR spectrum. The influence of the coupling effect can be demonstrated by Equation (1), as follows [29]:

(1) $$\lambda_{p}=\lambda_{metal}\sqrt{2n_{m}{}^{2}+1}$$

where λ_p_ is the LSPR peak wavelength, and λ_metal_ is the wavelength corresponding to the plasma resonance frequency of the bulk metal. n_m_ is RI of the surrounding medium. The lower resonance frequency was led by the weakened repulsive forces between two closed AuNSs, which generated interparticle plasma coupling effect. Consequently, the λ_metal_ increased because of its inversely proportional resonance frequency. The conclusion that higher λ_metal_ resulted in higher S_RI_ was drawn from Equation (1), with the red-shift of λ_p_.

### 2.2. Apparatus and Reagents

PAH (poly(allylamine hydrochloride)) was bought from Sigma-Aldrich. Sinopharm Chemical Reagent Beijing Co., Ltd. (Beijing, China) provided the KOH, ethanol, glycerol, and dextrose. TedPella provided the AuNS colloid solutions. All the reagents were analytic grade. Notably, all processes were carried out at room temperature. The trials utilized deionized water from the Milli-Q system (Millipore).

**Figure 1:**
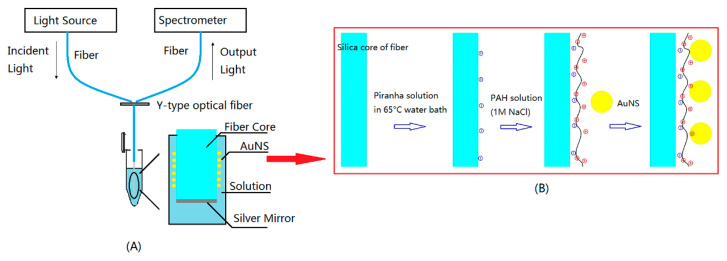
Schematic of (**A**) the fiber sensor design and (**B**) the fabricating processes of AuNS.

The SEM of sensing substrate was acquired by S-4800 (HATACHI). Thorlabs supplied multimode optical fiber (600 μm, NA = 0.37). Figure 1A shows the design of detecting system. A light source (DT- MINI-2-GS, Ocean Optics) guided white light into the fiber sensor, which attached to a split optical fiber. The light reflected by silver mirror at terminal of sensor was captured by a spectrometer (QE65pro, Ocean Optics) and created the LSPR spectrum.

### 2.3. Pretreatment

A stripping tool was used to remove the polymer cladding off the optical fiber. The inner sheathing of the fiber was etched with hot concentrated H_2_SO_4_, (98%) in beaker by alcohol lamp heating for 15 min until the sheathing fell off after carbonization. After thorough cleaning by DI water, the fiber’s two ends were polished by standard polishing process. Unclad sections of the fiber were treated with a Piranha solution (volume ratio 3:7 for H_2_O_2_ and H_2_SO_4_) for 1 h. The fiber was cleaned with DI water and then baked for 1 h at 110 °C.

### 2.4. AuNSs Immobilization

Figure 1B shows that electrostatic self-assembly was used to chemically modify the optical fiber in PAH (2 mg/mL, 1 M NaCl) for 2 h to form positive layer. After thorough rinsing by DI water, the unclad fiber was immobilized by AuNS after submerged in AuNS colloid with encapsulated citrate. A thin silver mirror on end face was prepared by the following steps. A regent mixed by 0.1 M AgNO_3_, 2% ammonia, and 0.8 M KOH was shocked with 0.05 M glucose solution. Finally, after dipping into the solution for 10 min, the end face was covered by silver mirror.

### 2.5. S_RI_ Tests

To evaluate the performance for RI detection, a series of different RI solutions, from 1.3322 to 1.4045, were used by preparing different concentrations of glycerol aqueous solutions in Table 1. The concentrations of glycerol solutions and their RI were determined using an Abbe Refractometer at 25 °C. LSPR fiber sensors were used to evaluate S_RI_ by the glycerol solutions with low to high RI successively. The fiber sensor was submerged in each sample until the LSPR spectrum stabilized. Spectrasuite (Ocean Optics) was used to record the data. The fiber sensor was completely cleaned after each test.

## 3. Results

**Figure 2:**
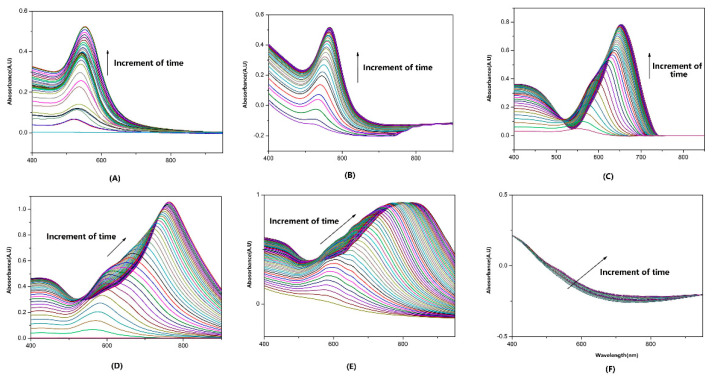
The variety of absorbance spectra during the (**A**) 20 nm, (**B**) 40 nm, (**C**) 60 nm, (**D**) 80 nm, (**E**) 100 nm, and (**F**) 150 nm AuNS immobilizing processes.

The LSPR spectra changed in the AuNS immobilizing process. To evaluate the different sizes of AuNS immobilizing processes at the surface of the fiber, the silver mirror was fabricated before the AuNS adsorption. Then, after dipping the fibers into the AuNS solutions with different sizes, the absorbance of the LSPR spectrums were recorded at 1 min intervals. As Figure 2A–F shows, with the increasing amount of AuNS, both the absorbance intensity and resonance wavelength of the LSPR spectrums were increased, except for 150 nm. It indicated that 150 nm AuNS was hard for the fiber to adsorb by the electrostatic attractive force because of its large particle size. For different sizes of AuNS-modified fiber sensors, all of their spectrums had rapid intensity increases in the initial 20 min and reached a state of stabilization after 60 min, which meant the saturated immobilization of AuNS. The baseline of the spectrum had some fluctuations, and even positive absorbance, which was led by the light resource and light path, and the LSPR peak position was impacted slightly. In addition, with the adsorbing of AuNS, the λ_p_ had a red shift, which was obviously enhanced with the increment size of AuNSs. This red shift indicated that the coupling effect between the approaching isolated AuNS was enhanced significantly. Therefore, we have reasons to expect the achievement of a high S_RI_ LSPR fiber sensor.

### 3.1. Effect of AuNS Size

Five different sizes (20 nm to 100 nm) of AuNS were utilized to examine the influence of size on the S_RI_ operating in wavelength-modulated modes. Various sizes of AuNSs were coated on the surface of the fiber for 60 min. After complete cleaning, the fibers were immersed in the various glycerol solutions (RI: 1.3322 to 1.3664). The S_RI_ was stated in terms of the shift of the peak per RI unit (nm/RIU), as defined by Equation (2):

(2) $$S=\frac{\delta \lambda_{p}}{\delta n}$$

**Figure 3:**
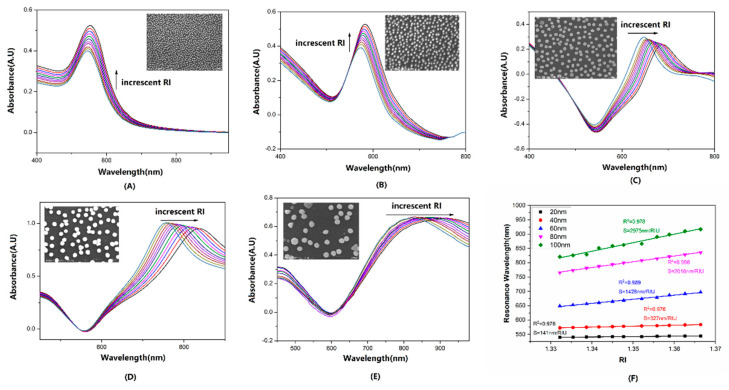
The absorbance spectrum series of optical fibers coated with (**A**–**E**) 20 nm to 100 nm AuNS in glycerol aqueous solutions with RI over the range from 1.3322 to 1.3664. (**F**) The S_RI_ and R-squared of linear fitting for RI and λ_p_.

Matlabs estimated λ_p_ using the weighted centroid algorithm [30]. Figure 3A,B demonstrated that, for 20 nm and 40 nm AuNSs, the LSPR spectrums with higher RI displayed a significant rise in absorbance intensity. λ_p_ had redshifts of 4.82 and 11.18 nm, respectively. Then, as seen in Figure 3C–E, the LSPR spectrums from 60 nm to 100 nm AuNSs with increasing RI mainly displayed the red shift of λ_p_. Even at 60 and 80 nm, the absorbance of the resonance wavelength was clearly reduced. The insets of Figure 3A–E show SEM images of the AuNS distribution. The SEM pictures revealed that all of the immobilized AuNSs of various sizes exhibited excellent dispersity and density. The maximum S_RI_, approximately 2975 nm/RIU, was measured by 100 nm AuNS. However, the peak line width was broadened with the increasing sizes because of the multipolar excitations, radiative damping, and interparticle plasmon coupling. This broadening of the spectral lines, which led to the accuracy loss of determining λ_p_, needs to be considered in biochemical detection. Figure 3F indicates that the S_RI_ had a significant improvement with the increased size of AuNSs. Additionally, Figure 3F shows that the λ_p_ in the same RI surrounding (DI water) was raised from 539.18 nm to 820.84 nm for 20 nm to 100 nm-AuNS, respectively. All of the aforementioned phenomena were consistent with Equation (1).

### 3.2. Effect of AuNS Density

**Figure 4:**
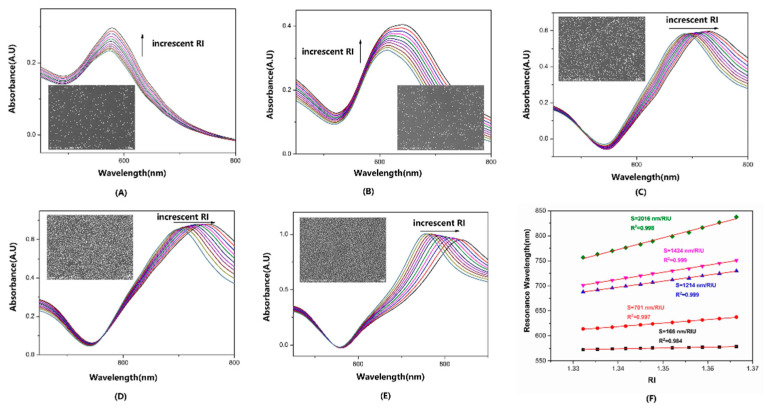
The absorbance spectrum series in glycerol solutions of fiber sensors immersed into 80 nm AuNS colloid for (**A**–**E**) 2, 5, 15, 30, and 60 min. (**F**) The S_RI_ and R-squared of fiber sensors with different AuNS densities.

Coating periods were varied from 2 min to 60 min to evaluate the influence of AuNS density on the performance of 80 nm AuNS-modified fiber sensors. According to Figure 4A–E, the distinct RI media were distinguished by different colors. The SEM images with different densities were shown in the inset of Figure 4A–E. With the density increased, the LSPR spectrums were changed with the increscent RI from increased intensity to redshift of λ_p_. As Figure 4F shows, the S_RI_, which increased gradually from 246 nm/RIU (lowest density) to 2016 nm/RIU (highest density), indicated that S_RI_ was closely related to the AuNS density. Additionally, the increasing density led to the raise of absorbance.

### 3.3. Effect of Sensing Length

**Figure 5:**
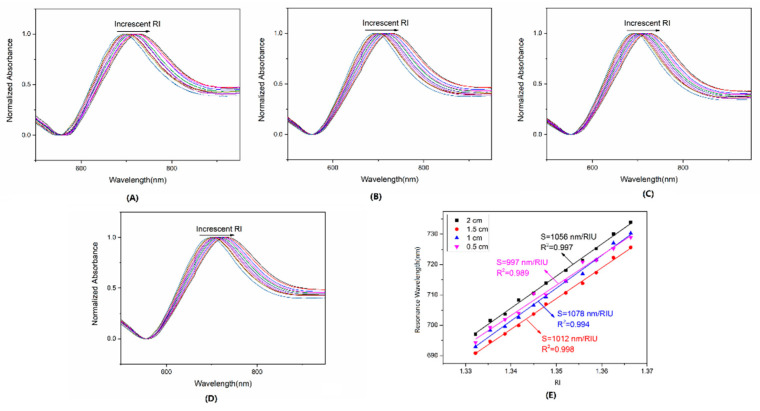
The normalized absorbance spectrum series of fiber sensors in glycerol solutions with different lengths for (**A**–**D**) 0.5, 1, 1.5, and 2 cm, and (**E**) the linear fitting curves of RI vs. λ_p_ with different lengths.

An 80 nm-AuNS immobilized fiber with an incubating time of about 15 min was tested for four different lengths ranging from 0.5 to 2 cm in different RI solutions (1.3322–1.3664). Figure 5A–D were the normalized LSPR spectrums of different sensing lengths. With the increasing of the RI medium, the λ_p_ of four figures appeared to red shift significantly. As Figure 5E shows, the S_RI_ for 0.5, 1, 1.5, and 2 cm were 997, 1078, 1012, and 1056 nm/RIU, respectively, with a relative standard deviation (RSD) of about 3.6%. The above demonstrated that the sensing length of the fiber was not associated with wavelength mode S_RI_. Furthermore, the result demonstrated that the S_RI_ was decided by 80 nm AuNS density, which determined the strength of the local plasma coupling.

### 3.4. Effect of RI Range

**Figure 6:**
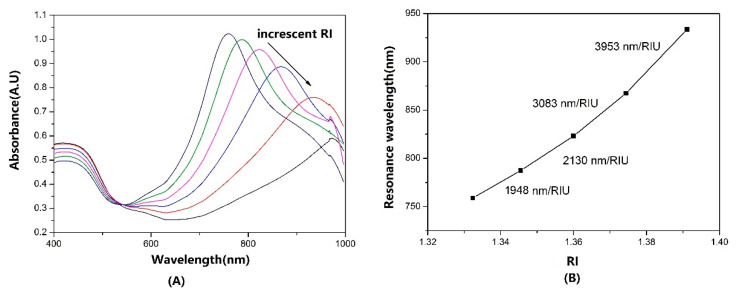
(**A**) The absorbance spectrums of 80 nm AuNS–modified fiber sensors in glycerol solutions, with RI range: 1.3322 to 1.4045. (**B**) The nonlinear increased S_RI_ for RI range: 1.3322 to 1.3911.

From Equation (1), it can be seen that the λ_p_ and RI only showed an approximately linear relationship in a narrow RI range, and the 80 nm AuNS-modified fiber optic sensor was dipped into different glycol solutions from 0 to 50%, with 10% intervals (RI range: 1.3322 to 1.4045). The LSPR spectrums are shown in Figure 6A. The λ_p_ was raised from 765.56 nm to 933.49 nm. In the solution with RI: 1.4045, the λ_p_ extended to over 980 nm, which was out of the spectral range of the spectrometer. In Figure 6B, the RI sensitivity showed a nonlinear increase with increasing RI. The highest S_RI_ reached 3953 nm/RIU, which had similar measurement capabilities, compared to SPR. The nonlinear growth tendency of S_RI_ was consistent with Equation (1).

### 3.5. Enhanced Sensitivity Analysis

**Figure 7:**
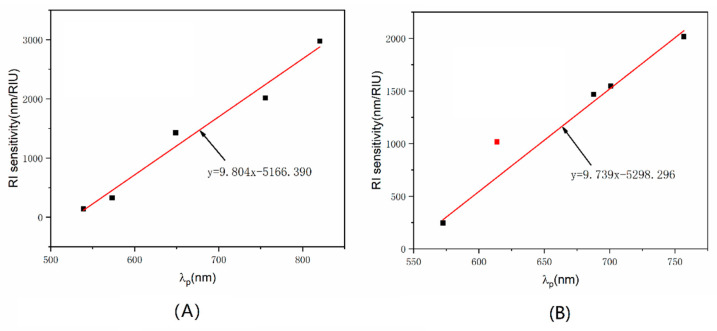
Linear fitting between S_RI_ and λ_p_ for (**A**) different sizes and (**B**) different densities (red square point outlier did not participate in the fitting).

The enhanced S_RI_ has been explained by Equation (1) in Section 2.1. In addition, the measurement results of RI by different sizes or densities of AuNS were used to support the above explanation. The S_RI_ of the LSPR fiber sensors with different sizes or densities and λ_p_ in deionized water were fitted linearly, respectively. As shown in Figure 7A, the S_RI_ and λ_p_ (n_m_ = 1.3322) were approximately linear for different AuNS sizes (Section 3.1), with S_RI_ = 9.804λ_p_ − 5166.390 (R^2^ = 0.969). In Figure 7B, the linear fitting equation for different densities (Section 3.2) was S_RI_ = 9.739λ_p_ − 5298.296(R^2^ = 0.992). From the fitting results, the two equations had similar parameters, which consistently indicated the intrinsic linear relationship between S_RI_ and λ_p_. According to Equation (1), λ_metal_ and λ_p_ were linearly related when nm was constant. Therefore, the S_RI_ and λ_metal_ was the linear relationship that confirmed that high S_RI_ was determined by the interparticle plasma coupling effect.

### 3.6. Figure of Merit (FOM)

The sensing capabilities for RI changes were dependent on the S_RI_. Additionally, the resonance peak linewidth should be considered because the broadening of resonance linewidth caused the accuracy reduction of λ_p_. The sensitivity of larger AuNS are typically higher, but their peaks are widened by multipolar excitations and radiative damping. A figure of merit (FOM), given by Equation (3), is often used to assess the detecting capabilities of a wavelength mode LSPR sensor [31].

(3) $$FOM=\frac{S}{\mathrm{FWHM}}$$

Here, FWHM was the full width at half-maximum of the LSPR spectrum. Equation (3) was used for isolated nanoparticles. It was challenging to determine a consistent FWHM for AuNP monolayer plasmonic nanostructures. FOM*, rather than FOM, is described by the maximum value of relative intensity change dI/I at a given wavelength for a tiny change d_n_ [32].

(4) $$FOM^{*}={\left(\frac{\frac{dI}{dn}}{I}\right)}_{\max}={\left(\frac{S\frac{dI}{d\lambda }}{I}\right)}_{\max}$$

The FOM*, which was calculated by Equation (4) for different sizes of AuNS-coated fiber sensors, are listed in Table 2. As Table 2 shows, though the S_RI_ increased with AuNS size, the 60 nm AuNS had the highest FOM*, about 26.47. It indicates that S_RI_ was not in keeping with FOM*, for the reason that the FWHM of the LSPR peak was significantly broadened with the increasing of the AuNS size. In addition, the enhanced coupling effect between the isolated AuNS also led to the broadening of FWHM.

### 3.7. Consistency and Stability

**Figure 8:**
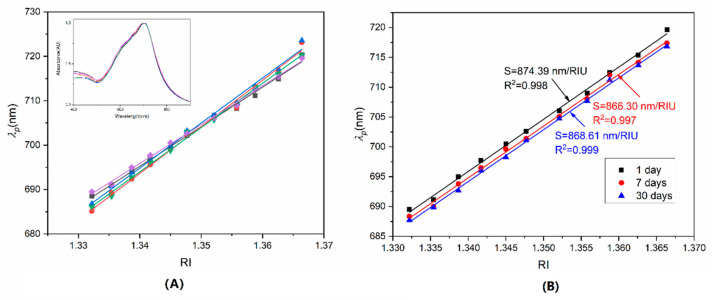
(**A**) S_RI_ of five fiber sensors for consistency test, and (**B**) S_RI_ of three test at 1 day, 7 days, and 30 days for stability evaluation.

The consistency was evaluated by RI test using five 80 nm AuNS-modified optical fiber sensors, which were fabricated simultaneously. Each fiber sensor was tested in the RI range from 1.3322 to 1.3664, after thorough cleaning with deionized water. The S_RI_ were about 891.92, 1049.62, 1014.25, 996.96, and 874.39 nm/RIU, respectively, in Figure 8A. The RSD was about 8.05%. Additionally, the inset in Figure 8A was the LSPR spectrums of five sensors in the same RI solution, about 1.3322. The λ_p_ were 688.50, 686.03, 686.78, 685.12, and 687.56 nm, with an RSD of about 0.19%. The test results indicated that the LSPR fiber sensors had good consistency. The stability was verified by one 80 nm AuNS-coated fiber, which was tested in the RI solutions three times, at 1 day, 7 days, and 30 days. The linear fitting curves between RI and λ_p_ are shown in Figure 8B. The S_RI_ in the three tests were 874.39, 866.30, and 868.61 nm/RIU, with an RSD of about 0.48%. The results indicated that this LSPR fiber sensor had great stability.

### 3.8. Performance Comparison

The major and influential literature and data of the wavelength mode of LSPR fiber sensors are listed in Table 3. From Table 3, we can see the key parameters about S_RI_ that have been studied for more than ten years. Some groups used U-bend, D-type, or tapered fiber to increase the interaction distance between the light and metal nanoparticles through evanescent fields. More groups improved the S_RI_ by changing the sharps of the metal nanoparticles. For the modified method, silane coupling agents, such as APTMS and MPTMS, were mainly used, even for time-consuming and uneven adsorption. Using the simplest materials, such as AuNS, multimode fiber, and PAH, the LSPR fiber sensor in this work showed significantly high S_RI_ and regular LSPR absorption spectral lines.

## 4. Conclusions

In summary, a simple and speedy pretreatment approach created a high-performance LSPR fiber sensor, without etching, bending, or tapering of the silica core. The fabricating process, in which the total time was only less than 4 h, has just five main detailed steps: fiber stripping, hydroxylation, PAH coating, AuNS adsorption, and retroreflector formation. After the fabricating process, several key factors, such as size, density, sensing length, and RI range, were optimized by RI measurements. The primary four conclusions can be drawn: (1) Under the condition of the thorough adsorption of AuNS, the LSPR fiber sensors modified by larger AuNS size led to the higher S_RI_. (2) Higher S_RI_ was achieved by increasing the AuNS density. (3) Under the condition of the same AuNS density, changes of sensing length did not have a significant relation to the S_RI_. (4) The enhanced S_RI_ analysis indicated that high S_RI_ was mainly attributed to the plasmon coupling effect between the isolated AuNS. Compared to the other wavelength mode LSPR fiber sensors reported, much higher S_RI_, up to 2975 nm/RIU and 3953 nm/RIU, were achieved by different sizes of AuNS-modified optical fibers in different RI ranges. Then, FOM* was raised, as the complementary parameter, to evaluate the performance of the LSPR fiber sensor further. For FOM*, 60 nm AuNS was the optimal parameter. Additionally, the sensor was proven to have good consistency and stability over time. Finally, this easily fabricated, high-performance sensor can improve the detection capability of biochemical LSPR fiber sensors. Moreover, its high-density and good dispersity will promote the application of SERS detection.

## Figures and Tables

**Table 1:** The concentration of test solutions and corresponding values of RI.

Concentration of Glycerol Solutions	RI
0%	1.3322
2%	1.3354
4%	1.3387
6%	1.3417
8%	1.345
10%	1.3477
12%	1.3521
14%	1.3558
16%	1.3588
18%	1.3626
20%	1.3664
30%	1.3744
40%	1.3911
50%	1.4045

**Table 2:** FOM* of fiber sensors with different AuNS sizes.

AuNS Size (nm)	20	40	60	80	100
Sensitivity (nm/RIU)	141	330	1428	2016	2975
FOM*	0.64	8.40	26.47	19.74	1.10

**Table 3:** Comparisons of SRI of LSPR fiber sensors by literature data.

Nanostructure	Modified Method	Type of Fiber	Sensitivity (nm/RIU)	Year	Ref.
Gold nanospheres	APTMS	multimode fiber	154–914	2012	[20]
Gold nanorod	MPTMS	multimode fiber	601	2013	[19]
Gold nanostar	MPTMS	tapered fiber	1190.5	2012	[15]
Gold nanostar	Laser deposition	D-type PMMA fiber	580	2013	[18]
Gold nanocages	APTMS	multimode fiber	1933	2014	[21]
Silver nanospheres	APTMS	multimode fiber	387	2015	[14]
Silver nanospheres and graphene	Laser deposition	U-bend multimode fiber	1198	2017	[22]
Gold nanospheres	PS-b-P4VP	multimode fiber	1079	2019	[23]
Gold nanorod	PS-b-PAA	multimode fiber	753	2020	[24]
Gold nanospheres& multilayer graphene	PAH	U-bend multimode fiber	1251.44	2020	[17]
Gold nanospheres	APTES	tapered fiber	664.66	2021	[13]
Gold nanospheres	PAH-PSS	multimode fiber	1312	2022	[25]
Gold nanospheres	PAH	multimode fiber	2975	2022	This work

## Data Availability

Data and information developed in this research are available.
